# Association between the histopathological growth patterns of liver metastases and survival after hepatic surgery in breast cancer patients

**DOI:** 10.1038/s41523-020-00209-1

**Published:** 2020-12-18

**Authors:** Ali Bohlok, Peter Vermeulen, Sophia Leduc, Emily Latacz, Lara Botzenhart, François Richard, Maxim De Schepper, Tatjana Geukens, Valerio Lucidi, Michail Ignatiadis, Philippe Aftimos, Christos Sotiriou, Martine Piccart, Alain Hendlisz, Steven Van Laere, Luc Dirix, Jean-Christophe Noël, Elia Biganzoli, Denis Larsimont, Christine Desmedt, Vincent Donckier

**Affiliations:** 1 Department of Surgery, Institut Jules Bordet, Université Libre de Bruxelles, Brussels, Belgium; 2 Translational Cancer Research Unit, GZA Hospitals & CORE, MIPRO, University of Antwerp, Antwerp, Belgium; 3 Department of Oncological Research, Oncology Center, GZA Hospitals Sint-Augustinus, Antwerp, Belgium; 4 Laboratory for Translational Breast Cancer Research, Department of Oncology, KU Leuven, Leuven, Belgium; 5 Department of Abdominal Surgery, Hôpital Erasme, Université Libre de Bruxelles, Institut Jules Bordet, Université Libre de Bruxelles, Brussels, Belgium; 6 Medical Oncology Clinic, Institut Jules Bordet, Université Libre de Bruxelles, Brussels, Belgium; 7 Clinical Trials Conduct Unit, Institut Jules Bordet, Université Libre de Bruxelles, Brussels, Belgium; 8 Breast Cancer Translational Lab, Institut Jules Bordet, Université Libre de Bruxelles, Brussels, Belgium; 9 Medical Oncology Department, Institut Jules Bordet, Université Libre de Bruxelles, Brussels, Belgium; 10 Department of Pathology, Hôpital Erasme, Université Libre de Bruxelles, Brussels, Belgium; 11 Department of Clinical Sciences and Community Health & DSRC, University of Milan, Fondazione IRCCS Istituto Nazionale Tumori, Milan, Italy; 12 Department of Pathology, Institut Jules Bordet, Université Libre de Bruxelles, Brussels, Belgium

**Keywords:** Breast cancer, Prognostic markers

## Abstract

Currently, there are no markers to identify patients with liver-only or liver-dominant metastases that would benefit from hepatic surgery. Here we characterized histopathological growth patterns (HGPs) of liver metastases in a consecutive series of 36 breast cancer patients who underwent hepatic surgery. Survival analyses showed that the presence of a desmoplastic HGP in the liver metastases (a rim of fibrous tissue separating cancer cells from the liver parenchyma, present in 20 (56%) patients) is independently associated with favorable progression-free and overall survival when compared with the replacement HGP (cancer cells growing into the liver parenchyma, present in 16 (44%) patients).

Even if metastatic breast cancer is frequently considered as a systemic disease, local treatment targeting metastases may result in prolonged survival in selected cases^1–4^. In particular, in patients with liver-only or liver-dominant metastases, surgical resection of liver metastases (LM) is associated with survival between 22 and 61 months and long-term progression-free survival (PFS) in selected cases^4^, serving as a proof of concept for an oligometastatic status in a subgroup of patients. At present, however, there are no established biomarkers to identify this subgroup^5^. Furthermore, no factor has been reliably associated with rapid postoperative recurrence, and consequently, a substantial proportion of patients operated for breast cancer LM undergo futile and possibly even detrimental surgery. Accordingly, the role of surgery for treatment of breast cancer LM remains largely debated^6^.

In large retrospective studies, the histopathological growth patterns (HGPs) of resected LM of patients with colorectal cancer predict the postoperative outcome, clearly surpassing the prognostic power of the traditional clinical risk scores^7–9^. HGPs are identified by light microscopy in standard hematoxylin-and-eosin-stained (H&E) tissue sections at the interface between the tumor and the liver (Fig. 1a, b). International consensus guidelines for HGP scoring have been established, allowing reproducible and accurate assessment of the HGP of LM.^10^ Due to the heterogeneity of the HGP within a metastasis, this can only be reliably assessed on surgical resection specimen and not on core needle biopsies. In the replacement pattern (R-HGP), cancer cells infiltrate the hepatic plates and replace the resident hepatocytes, thereby co-opting the sinusoidal blood vessels of the liver. In metastases with a desmoplastic pattern (D-HGP), cancer cells are separated from liver cells by a rim of desmoplastic stroma, which is often densely infiltrated with inflammatory cells. In the D-HGP, tumor vascularization is provided by angiogenesis. In colorectal patients undergoing resection of LM, the R-HGP is associated with worse postoperative survival as compared to D-HGP LM^7–9^. Here we aimed at scoring and evaluating the prognostic value of HGP in patients with breast cancer undergoing resection of LM.

**Fig. 1:**
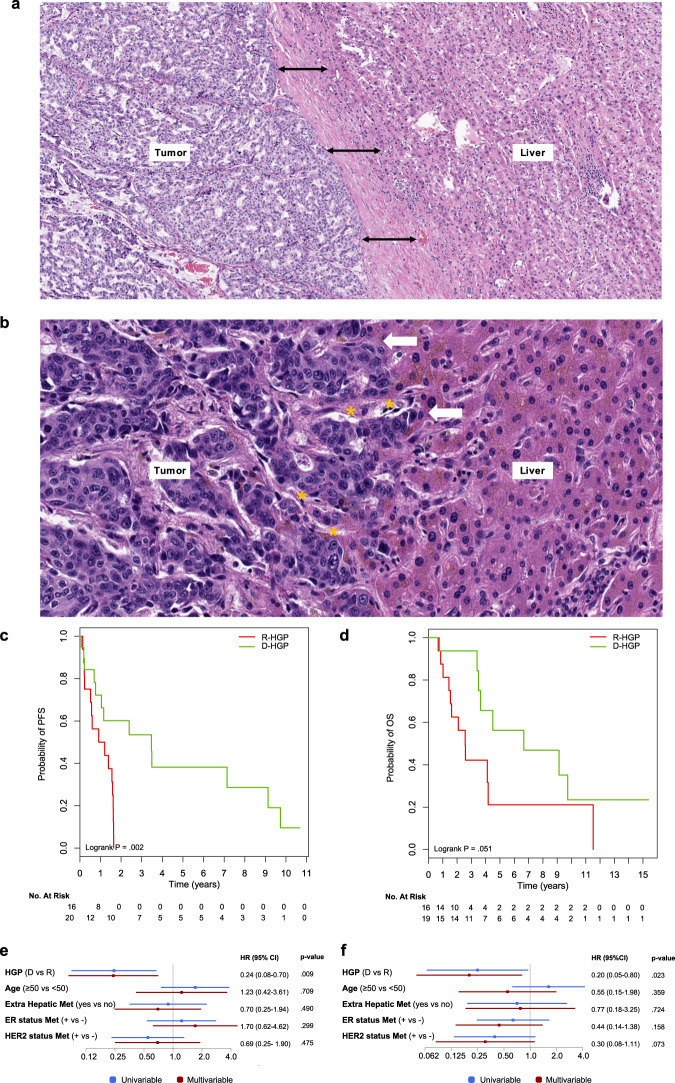
Histological growth patterns in liver metastases from breast cancer patients. **a** Breast cancer LM with a desmoplastic growth pattern (H&E staining): black double-headed arrows indicate the desmoplastic rim that separates the tumor tissue from the liver parenchyma. **b** Breast cancer liver metastasis with a replacement growth pattern (H&E staining): the white arrows indicate some of the regions where cancer cells grow into the liver cell plates and replace the hepatocytes. Cancer cells are in contact with the hepatocytes. The yellow asterisks mark two co-opted sinusoidal blood vessels. **c** Kaplan–Meier curves displaying the PFS probability according to the HGP group. **d** Kaplan–Meier curves displaying the OS probability according to the HGP group. **e** Univariate and multivariate Cox regression analyses for PFS. **f** Univariate and multivariate Cox regression analyses for OS. CI confidence interval, HGP histological growth pattern (D desmoplastic, R replacement), H&E hematoxylin and eosin, LM liver metastasi(e)s, PFS progression-free survival, OS overall survival.

Consecutive patients with breast cancer who underwent surgical resection for LM at the Institut Jules Bordet and the Hôpital Erasme (Brussels, Belgium) between April 2000 and October 2017 were included in the study. This resulted in 36 patients with a median follow-up of 10.7 years (Table 1). A total of 175 slides were evaluated for the LM from these 36 patients (median = 4 and average = 4.9, Supplementary Fig. 1). The distribution of the percentages of the R-HGP and D-HGP components are represented per patient in Supplementary Fig. 2a. Of note, we did not observe a correlation between the number of slides that were evaluated per patient and the percentage of R-HGP (Supplementary Fig. 2b). For 11 patients, the HGPs were evaluated in multiple metastases. In agreement with what has already been reported in LM from patients with colorectal cancer^11^, we observed a low intra-patient inter-metastasis heterogeneity with regard to the HGP (Supplementary Fig. 3). We further categorized the LM based on their HGP for the remaining analyses: (1) LM with a pure replacement HGP (i.e. present in 100% of the tumor–liver interface in all the available sections), further referred to as “pure R-HGP” and present in 16 patients (44%) and (2) LM that are at least partly (1% of interface or more) desmoplastic, further referred to as “any D-HGP” and present in the remaining 20 patients (56%).

**Table 1:** Patient and sample characteristics according to the histological growth pattern present in the liver metastasi(e)s.

		Pure R-HGP	Any D-HGP	*p* (Fisher)
Menopausal status at primary diagnosis	Pre	6 (40.0)	13 (76.5)	0.070
	Post	9 (60.0)	4 (23.5)	
	Missing	1	3	
Age at primary diagnosis	≤50	6 (37.5)	12 (60.0)	0.315
	>50	10 (62.5)	8 (40.0)	
TNM_T	0	0 (0.0)	1 (6.2)	0.172
	1	6 (46.2)	10 (62.5)	
	2	6 (46.2)	3 (18.8)	
	3	0 (0.0)	2 (12.5)	
	4	1 (7.7)	0 (0.0)	
	Missing	3	4	
TNM_N	0	3 (21.4)	10 (55.6)	0.020
	1	8 (57.1)	2 (11.1)	
	2	3 (21.4)	6 (33.3)	
	Missing	2	2	
TNM_M	0	9 (56.2)	14 (70.0)	0.493
	1	7 (43.8)	6 (30.0)	
Grade (primary)	1	5 (35.7)	2 (12.5)	0.356
	2	5 (35.7)	9 (56.2)	
	3	4 (28.6)	5 (31.2)	
	Missing	2	4	
ER status (primary)	Negative	3 (21.4)	2 (12.5)	0.642
	Positive	11 (78.6)	14 (87.5)	
	Missing	2	4	
PgR status (primary)	Negative	6 (42.9)	5 (31.2)	0.707
	Positive	8 (57.1)	11 (68.8)	
	Missing	2	4	
HER2 status (primary)	Negative	8 (61.5)	10 (62.5)	1
	Positive	5 (38.5)	6 (37.5)	
	Missing	3	4	
Histological type (primary)	IDC (NST)	12 (75.0)	16 (88.9)	0.410
	ILC	2 (12.5)	2 (11.1)	
	Other	2 (12.5)	0 (0.0)	
	Missing	0	2	
Neoadjuvant chemotherapy	No	7 (43.8)	13 (76.5)	0.080
	Yes	9 (56.2)	4 (23.5)	
	Missing	0	3	
Menopausal status at metastatic diagnosis	Pre	4 (28.6)	6 (35.3)	1
	Post	10 (71.4)	11 (64.7)	
	Missing	2	3	
Age at metastatic diagnosis	≤50	6 (37.5)	9 (45.0)	0.741
	>50	10 (62.5)	11 (55.0)	
ER status (met.)	Negative	6 (37.5)	7 (35.0)	1
	Positive	10 (62.5)	13 (65.0)	
PgR status (met.)	Negative	7 (43.8)	12 (60.0)	0.503
	Positive	9 (56.2)	8 (40.0)	
HER2 status (met.)	Negative	11 (68.8)	13 (65.0)	1
	Positive	5 (31.2)	7 (35.0)	
Nr hepatic met. lesions (preop.)	1	8 (50.0)	11 (55.0)	1
	>1	8 (50.0)	9 (45.0)	
Extra hepatic met.	No	12 (75.0)	16 (80.0)	1
	Yes	4 (25.0)	4 (20.0)	
Interval primary/met. (years)	<1 year	6 (37.5)	3 (15.0)	0.300
	1–5 years	5 (31.2)	10 (50.0)	
	>5 years	5 (31.2)	7 (35.0)	
ER status (P → LM)	Gain ER	1 (7.1)	1 (6.2)	0.934
	Loss ER	3 (21.4)	5 (31.2)	
	Stable ER−	2 (14.3)	1 (6.2)	
	Stable ER+	8 (57.1)	9 (56.2)	
	Missing	2	4	
PgR status (P → LM)	Gain PgR	3 (21.4)	1 (6.2)	0.622
	Loss PgR	3 (21.4)	6 (37.5)	
	Stable PgR−	3 (21.4)	4 (25.0)	
	Stable PgR+	5 (35.7)	5 (31.2)	
	Missing	2	4	
HER2 status (P → LM)	Gain HER2	1 (7.7)	1 (6.2)	1
	Loss HER2	2 (15.4)	2 (12.5)	
	Stable HER2−	7 (53.8)	9 (56.2)	
	Stable HER2+	3 (23.1)	4 (25.0)	
	Missing	3	4	
Size largest lesion	<50 mm	13 (81.2)	18 (90.0)	0.637
	≥50 mm	3 (18.8)	2 (10.0)	
Preoperative systemic treatment	No	1 (6.2)	1 (5.9)	1
	Yes	15 (93.8)	16 (94.1)	
	Missing	0	3	

*ER* estrogen receptor, *HGP* histological growth pattern (*D* desmoplastic, *R* replacement), *IDC* invasive ductal carcinoma, *ILC* invasive lobular carcinoma, *LM* liver metastasis, *NST* invasive carcinoma of no special type, *P* primary tumor, *PgR* progesterone receptor, *TNM* tumor–node–metastasis staging system.

There was no association between these HGP categories and estrogen receptor (ER) or HER2 status of the primary breast carcinoma or of the LM (Table 1). LM subtypes were as follows: 7 ER−/HER2− (19%), 12 HER2+ (33%), and 17 ER+/HER2− (47%). In 33% of the patients (10/30), the ER status differed between the primary tumor and LM. In 8 patients, ER expression was lost in the LM, while in 2 patients the LM gained ER expression. Significantly more patients in the “pure R-HGP” group had a primary tumor associated with lymph node metastases (79 versus 44% in the “any D-HGP” group; *p* = 0.02).

“Any D-HGP” was independently associated with better PFS after liver surgery when compared with “pure R-HGP” (adjusted hazard ratio (HR) = 0.24, 95% confidence interval (CI): 0.08–0.70; *p* = 0.009, Fig. 1c, e). All patients of the “pure R-HGP” group relapsed within the first 20 months after liver surgery. Similarly, improved overall survival (OS) was observed for patients with “any D-HGP” LM as compared to patients with “pure R-HGP” metastases (adjusted HR = 0.20, 95% CI: 0.05–0.80; *p* = 0.023, Fig. 1d, f).

In this study, we addressed whether the HGP has a potential to predict outcome in breast cancer patients undergoing surgical resection of LM. A first relevant finding is that a high fraction of these patients (44%) have LM with a pure R-HGP, whereas this pattern is only observed in 4–20% of the resected colorectal LM^7–9^. This indicates that the presence of the distinct HGPs may depend on the type of primary tumor. Comparable to what has been described for colorectal cancer^7–9^ and uveal melanoma^12^, we confirm the association between R-HGP and poor outcome after resection of LM. In contrast, D-HGP may thus identify breast cancer patients who can be offered (repeated) hepatic surgery to prolong survival. In this series, all patients with pure R-HGP LM rapidly relapsed within 2 years after surgery, indicating a more aggressive disease course and strongly questioning the role of surgery in these cases. If these results are confirmed in larger series, HGP assessment could be implemented in studies to test patient-tailored management of LM. Furthermore, if HGP could be predicted preoperatively, for example, by using dedicated medical imaging methods since it cannot be assessed on biopsies, it may represent a new factor for guiding the surgical decision in breast cancer patients with resectable LM. While D-HGP and R-HGP could present different radiological features, namely, at tumor–liver interface (see Supplementary Figs. 3 and 4 as an example), prospectively designed radiomics studies are needed to ensure adequate sensitivity and specificity of this approach^13^. Finally, there is a strong need for the molecular characterization of LM from breast cancer patients beyond the need for markers to guide the surgical decision. For instance, recent reports have suggested that immunotherapy based on checkpoint inhibition is less efficient in metastatic cancer patients with LM^14^. Although more studies are needed to evaluate the immune context in LM, this clinical observation could be related to the fact that LM with the D-HGP generally present the so-called immune-excluded phenotype and LM with the R-HGP the immune-desert phenotype^15,16^, as illustrated in Supplementary Fig. 5. Altogether, this study emphasizes the need for studying LM from breast cancer patients in more detail to allow further personalization of local and systemic treatment for these patients in the near future.

## Methods

### Scoring of the HGPs

The HGP of the LM was scored according to the international guidelines^10^ by an experienced pathologist (P.V.) blinded to the outcome data. All available H&E sections of all metastases were scored for each patient. The entire tumor–liver interface was evaluated for each tissue section. The HGP was scored as a relative proportion (percentage) of the interface in which each of the HGPs (replacement or desmoplastic) occurred. Average HGP scores were then calculated for each patient. Of note, we reported previously a high interobserver agreement for scoring HGP^17^.

### Statistical analyses

Clinical and pathological data were derived from the electronic patient files. Associations between HGP and clinicopathological characteristics were assessed with Fisher exact test. Associations with PFS and OS were assessed with Cox proportional hazard regression considering date of hepatic surgery as the starting time point, after assessing the proportional hazard assumptions. There were 29 and 20 events observed for PFS and OS, respectively. Age at hepatic surgery and ER and HER2 status of the LM as well as the presence of extra-hepatic metastases were considered as adjustment variables and center as stratification factor.

### Ethics approval

This study was approved by the ethical committee of both institutions (CE2953 on 5 March 2019 for Institut Bordet and P2019/232/NA on 4 April 2019 for the Hôpital Erasme) and the necessity for written informed consent was waived given the retrospective nature of the study.

### Reporting summary

Further information on research design is available in the Nature Research Reporting Summary linked to this article.

## Supplementary information

Supplementary Data

Reporting Summary

## Data Availability

The datasets that support the findings of this study are not publicly available but will be made available upon reasonable request, following ethics committee approval and a data transfer agreement, to guarantee the General Data Protection Regulation, as described in the following metadata record: 10.6084/m9.figshare.13177307.^18^ Please contact the corresponding author, V.D. (email address: vincent.donckier@bordet.be) to request access to the data.
