# Supplementary material for: Association between the histopathological growth patterns of liver metastases and survival after hepatic surgery in breast cancer patients
Source: NPJ Breast Cancer. 2020 Dec 18;6:64. doi: 10.1038/s41523-020-00209-1 (PMC7749172; doi:10.1038/s41523-020-00209-1)
Supplement: Supplementary file 1 — Supplementary Data [file 41523_2020_209_MOESM1_ESM.pdf]

SUPPLEMENTARY DATA: Association between the histopathological growth patterns of liver metastases and survival after hepatic surgery in breast cancer patients

Bohlok A. et al.

Contents

1. Supplementary methods 2
   1.1 Immunofluorescence staining 2
2. Supplementary Figures 2
3. References 6

1. Supplementary methods

1.1 Immunofluorescence staining

Two representative FFPE blocks were selected for replacement and desmoplastic growth pattern. Multiplex immunostaining with two markers in parallel was performed on 3micron sections, according to previous published protocol[1]. Hence the autofluoresence present in liver tissues, quenching of this autofluorescent signal was performed according to CycIF protocol[2]. The tissue section was first incubated by an unlabeled primary antibody, followed by incubation with a fluorophore labelled secondary antibody directed against the isotype of the primary antibody (see below). Finally, a nuclear counterstain was performed using DAPI. The slides were then scanned on AxioScan Z1 (Zeiss) with adapted scan profile.

Primary antibodies					Secondary antibodies
Protein	Clone	Species	Concentration	Company	
AE1/AE3	GA053	Mouse IgG1, kappa	0,4µg/mL	Dako	Alexa Fluor® 488
CD3	IR503	Polyclonal rabbit IgG	RTU	Dako	Alexa Fluor® 647

2. Supplementary Figures

Supplementary Figure 1: Histogram representing the number of slides per patient that were used for scoring the histopathological growth patterns

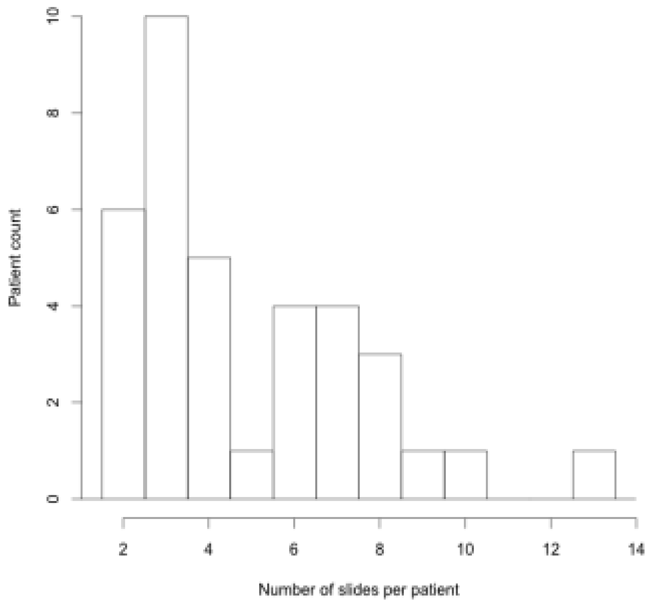

Supplementary Figure 2: Percentage of the histopathological growth patterns of the liver metastases of the 36 patients. a. Distribution of the percentage of the histopathological growth patterns. The ranking is based on the decreasing percentage R-HGP. Each bar represents the HGP assessment of one patient. **b.** Scatterplot representing the number of slides evaluated per patient versus the percentage of R-HGP. The black line represents the linear regression. The Spearman coefficient was 0.026 (p= 0.879).

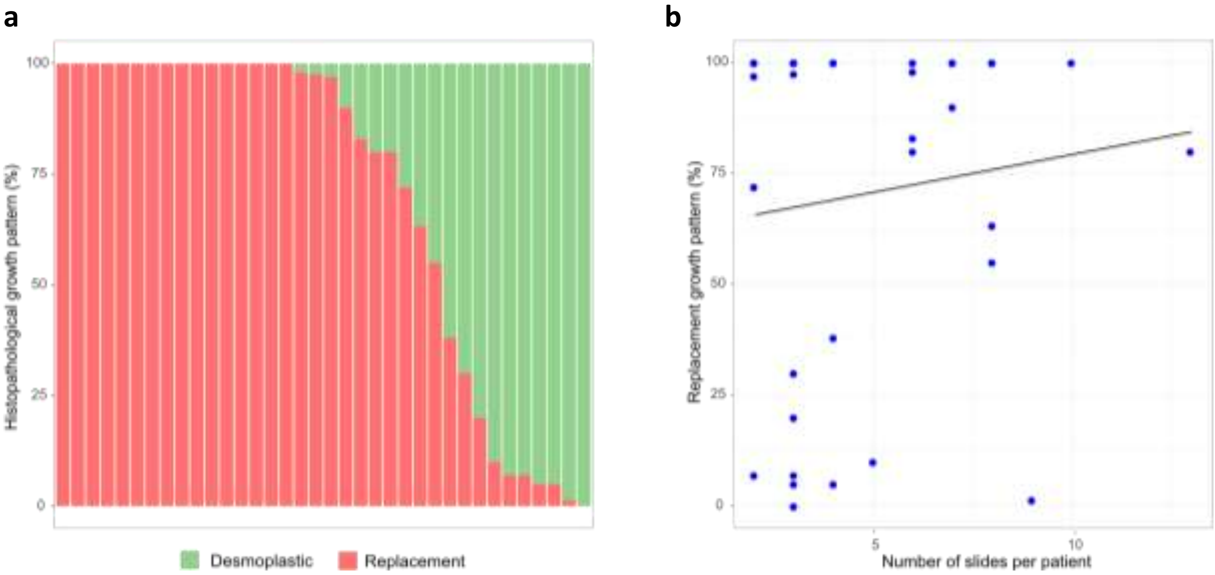

Supplementary Figure 3: Box-plot representation of the intra-patient inter-liver heterogeneity. Eleven patients had more than one liver metastasis that was examined after resection for the histopathological growth patterns. The blue dots represent the percentage of the replacement growth pattern of each metastasis and the red line the mean.

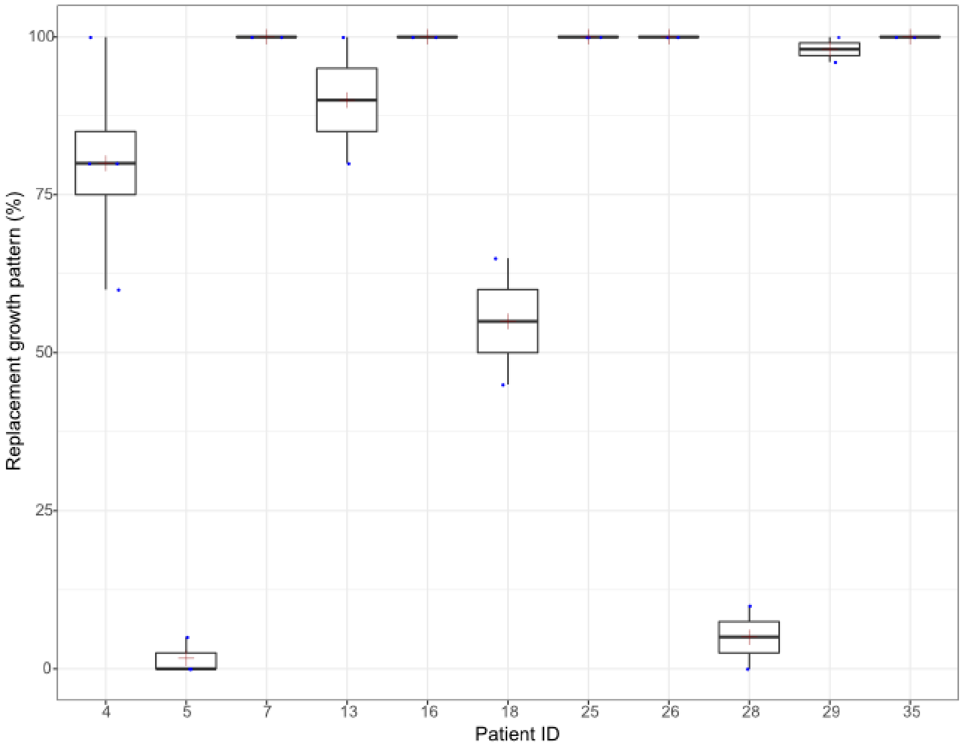

Supplementary Figure 4: Representative image of a liver metastasis presenting a desmoplastic growth pattern. Preoperative magnetic resonance imaging showing a well-encapsulated 14 mm liver metastasis in segment VI with a peri-tumor rim enhanced at portal phase (arrow). This patient had a pure desmoplastic histopathological growth pattern at pathological analysis.

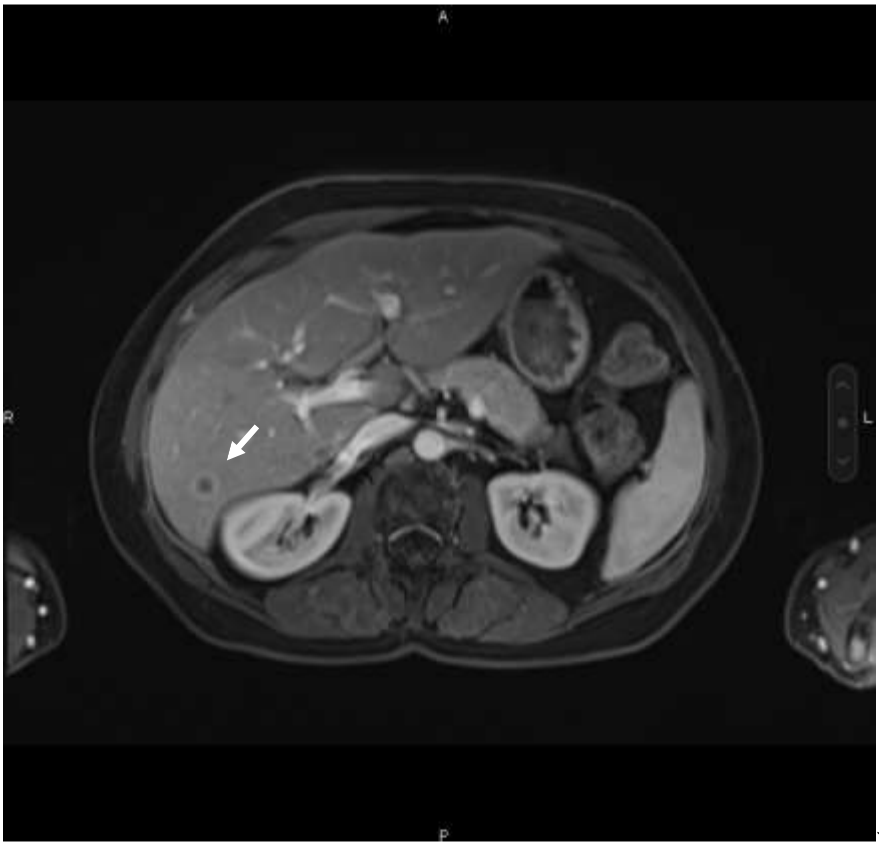

Supplementary Figure 4: Representative image of a liver metastasis presenting a replacement growth pattern. Preoperative magnetic resonance imaging showing an ill-defined 60 mm metastasis in segment VIII (white arrow) and inconstant peri-tumor rim enhancement at portal phase (red arrow). This patient had a pure replacement histopathological growth pattern on pathological analysis.

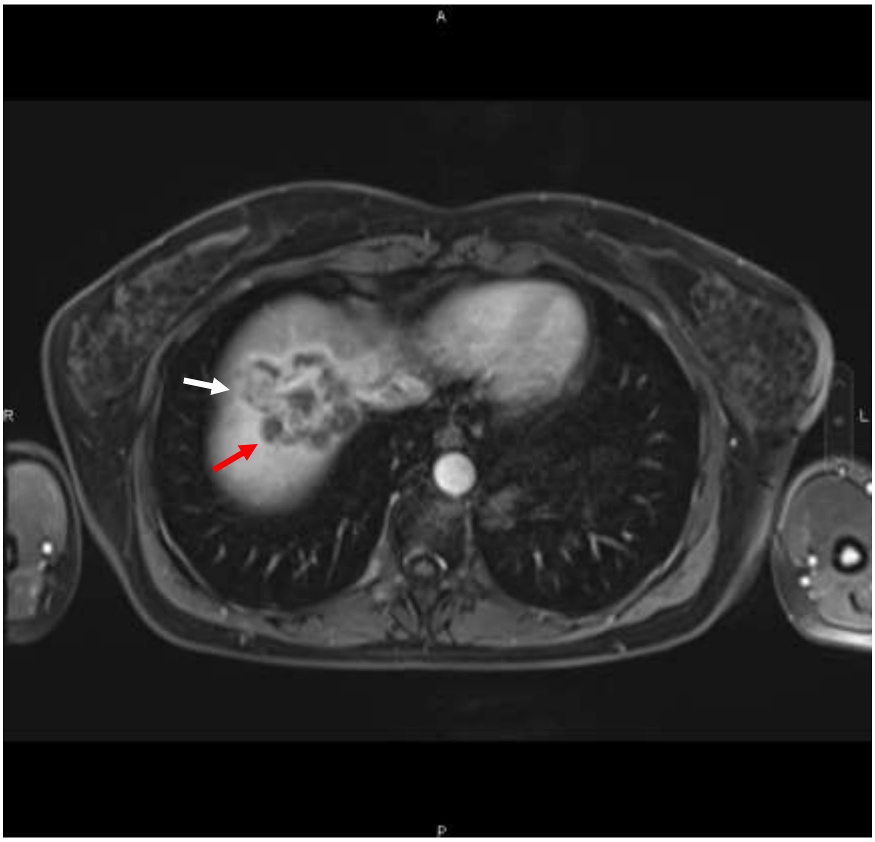

Supplementary Figure 5: Multiplex immunofluorescence image at invasive front of breast cancer liver metastasis (50X). Left: desmoplastic histopathological growth pattern. **Right:** replacement histopathological growth pattern. (AE1/AE3 displayed in green (cancer cells); CD3 displayed in red (T-lymphocytes), DAPI in blue (cell nuclei)).

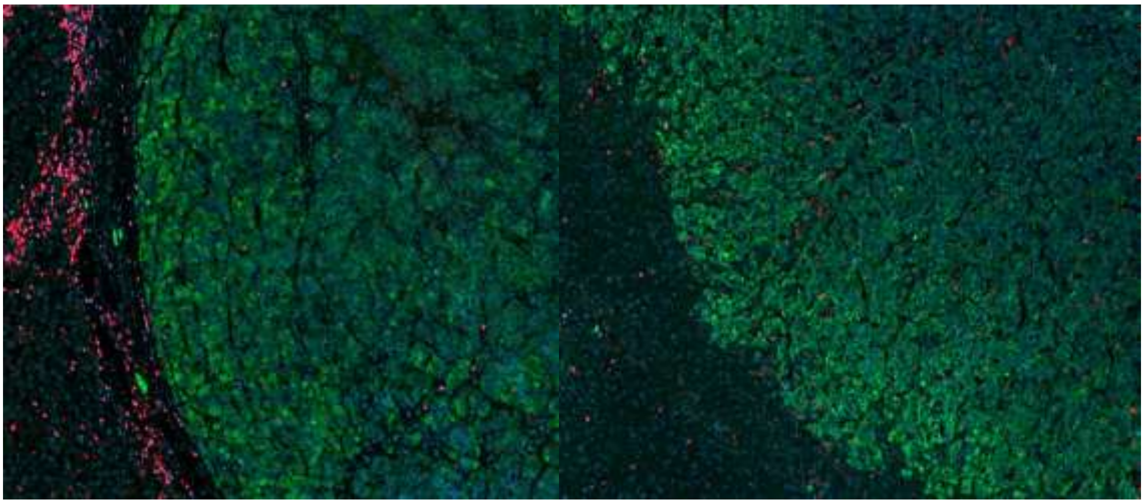

3. References

1. Bolognesi, M. M. *et al.* Multiplex Staining by Sequential Immunostaining and Antibody Removal on Routine Tissue Sections. *J. Histochem. Cytochem.* **65**, 431–444 (2017).
2. Lin, J. R., Fallahi-Sichani, M. & Sorger, P. K. Highly multiplexed imaging of single cells using a high-throughput cyclic immunofluorescence method. *Nat. Commun.* (2015).
